# First-Principles Assessment of ZnTe and CdSe as Prospective Tunnel Barriers at the InAs/Al Interface

**DOI:** 10.1021/acsami.4c17957

**Published:** 2025-01-13

**Authors:** Malcolm J. A. Jardine, Derek Dardzinski, Zefeng Cai, Vladimir N. Strocov, Moïra Hocevar, Christopher J. Palmstrøm, Sergey M. Frolov, Noa Marom

**Affiliations:** † Department of Materials Science and Engineering, Carnegie Mellon University, Pittsburgh, Pennsylvania 15213, United States; ‡ Swiss Light Source, Paul Scherrer Institut, CH-5232 Villigen PSI, Switzerland; § Univ. Grenoble Alpes, CNRS, Grenoble INP, Institut Néel, 38000 Grenoble, France; ∥ Materials Department, University of California-Santa Barbara, Santa Barbara, California 93106, United States; ⊥ ^∥^Department of Electrical and Computer Engineering, University of California-Santa Barbara, Santa Barbara, California 93106, United States; # Department of Physics and Astronomy, University of Pittsburgh, Pittsburgh, Pennsylvania 15260, United States; ¶ Department of Physics, Carnegie Mellon University, Pittsburgh, Pennsylvania 15213, United States; ∇ Department of Chemistry, Carnegie Mellon University, Pittsburgh, Pennsylvania 15213, United States

**Keywords:** density functional theory, electronic structure, metal−semiconductor interface, tunnel barrier, InAs, ZnTe, CdSe, Al

## Abstract

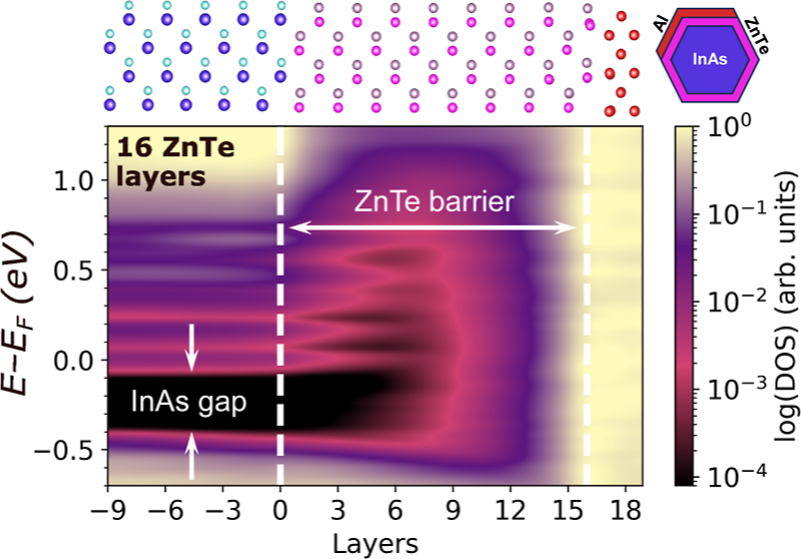

Majorana zero modes are predicted to emerge in semiconductor/superconductor interfaces, such as InAs/Al. Majorana modes could be utilized for fault tolerant topological qubits. However, their realization is hindered by materials challenges. The coupling between the superconductor and the semiconductor may be too strong for Majorana modes to emerge, due to effective doping of the semiconductor by the metallic contact. This could be mediated by adding a tunnel barrier of controlled thickness. We use density functional theory (DFT) with Hubbard U corrections, whose values are machine-learned via Bayesian optimization (BO), to assess ZnTe and CdSe as prospective tunnel barriers for the InAs/Al interface. The results of DFT+U(BO) for ZnTe are validated by comparison to angle resolved photoemission spectroscopy (ARPES). We then study bilayer interfaces of the three semiconductors with each other and with Al, as well as trilayer interfaces with a varying number of ZnTe or CdSe layers inserted between InAs and Al. We find that 16 atomic layers of either material completely insulate the InAs from metal induced gap states (MIGS). However, ZnTe and CdSe differ significantly in their band alignment, such that ZnTe forms an effective barrier for electrons, whereas CdSe forms a barrier for holes. Because of Fermi level pinning in the conduction band at the interface, only electron transport is relevant for InAs-based Majorana devices. Therefore, ZnTe is the better choice. Based on the results of our simulations, we suggest conducting experiments with ZnTe barriers in the thickness range of 6–18 atomic layers.

## Introduction

Majorana Zero Modes (MZMs) are an active area of research into non-Abelian quantum states, which could potentially offer a degree of protection from decoherence in quantum computing architectures.^1,2^ MZMs would theoretically manifest as zero-energy midgap states localized at boundaries and topological defects within one- and two-dimensional topological superconductors.^3^ One heavily researched platform for realizing MZMs involves proximity coupling a conventional superconductor, most commonly Al and Nb-based alloys, with a semiconductor nanowire with strong spin–orbit coupling (SOC), such as InAs or InSb. The realization of MZMs in such systems would require an external magnetic field or another form of broken symmetry.^4−7^ The current understanding is that initial experimental efforts did not, in fact, yield clear evidence of MZMs, but rather may have detected signatures that arose from disorder.^7−10^ This highlights the critical importance of materials selection, as well as the interface structure and quality, as paramount to eventually achieving MZMs. Recent advances in materials characterization and experimental techniques in semiconductor-nanowire/superconductor systems include investigating different combinations of semiconductors such as InAs and InSb and superconductors, such as Pb, Sn, Nb, aiming to improve electron mobility and harness larger superconducting gaps and higher critical magnetic fields.^11−14^

A challenge of the semiconductor/superconductor hybrid nanowire configuration is that excessive coupling between the superconducting metal and semiconductor can lead to the “metallization” of the semiconductor, which could hinder achieving the desired topological phase in experimental setups. Theoretical investigations, utilizing the Poisson-Schrödinger equation to model the semiconducting and superconducting properties of the system, have shown that excessive coupling leads to the renormalization of critical semiconductor properties, such as the Landé *g*-factor and spin–orbit-coupling (SOC), toward values of the metal. Furthermore, undesired band shifts may be induced.^11,15−22^ The incorporation of a tunnel barrier could serve to modulate the strength of the superconductor-semiconductor coupling, thereby influencing the induced proximity effect. Another benefit of a tunnel barrier is that it may act as a passivation layer on top of the semiconductor nanowire, protecting it from disorder and charge instabilities.^23−27^ Previously, we have explored this idea by using density functional theory (DFT) to investigate CdTe as a prospective tunnel barrier at the InSb/α-Sn interface.^28^ We found that 16 atomic layers (3.5 nm) of CdTe completely shield the InSb from metal induced gap states (MIGS) from the α-Sn, effectively decoupling the semiconductor from the metal. We therefore concluded that the thickness range most likely to be of interest for experimental exploration is 6–10 atomic layers, where some MIGS are still present, indicating coupling between the metal and semiconductor.

InAs and Al are prominent materials that are frequently used to fabricate nanowire based devices in search of MZMs.^15,27,29−32^ InAs is one semiconductor of choice in such setups thanks to its strong spin–orbit coupling and large Landé *g*-factor. Al, despite its relatively low transition temperature of 1.2 K, is compatible with established fabrication methods and has yielded devices with clear signatures of induced superconductivity. Al has been epitaxially grown on InAs nanowires producing well-ordered, domain-matched interfaces, in which the crystallinity of both the InAs and Al components was maintained. This was accompanied by the experimental observation of a hard superconducting gap induced in InAs.^12^ There has been one experimental study, in which an InGaAs tunnel barrier with varying thickness was inserted between InAs and Al.^27^ However, no first-principles studies of a tunnel barrier at the InAs/Al interface have been performed to date.

An ideal tunnel barrier should be a wide band gap semiconductor with a lattice constant that closely matches that of InAs to enable the growth of a high-quality epitaxial interface. In general, it is desirable for the band gap of a tunnel barrier to straddle the gap of the semiconductor in order to provide a barrier for both electrons and holes. However, in the case of InAs, because the Fermi level is pinned in the conduction band at the interface, and because electron mobility generally exceeds hole mobility, only electron transport is relevant. Hence a tunnel barrier for electrons is needed. ZnTe and CdSe have wide band gaps of 2.26 and 1.75 eV, respectively. The cubic zinc blende phases of InAs, ZnTe, and CdSe have similar lattice parameters with lattice mismatches of approximately 0.5% for InAs/ZnTe, 0.3% for InAs/CdSe, and 0.2% for ZnTe/CdSe.^33−38^ Based on their bulk band edge positions, the band gaps of both ZnTe and CdSe are expected to straddle the gap of InAs.^39,40^ Epitaxial growth of ZnTe and CdSe on InAs{100} and on each other, has been investigated in the context of visible wavelength semiconductor electronics,^36,41−43^ mid-IR lasers, spin-related phenomena,^44,45^ and as barriers in InAs-based heterostructures.^46−49^ Studies of the ZnTe/Al and CdSe/Al interfaces have aimed to elucidate the physics of metal–semiconductor interfaces in the context of devices such as light emitting diodes (LEDs) and Schottky diodes, where the Fermi level position and interface structure are of importance.^50−54^ Therefore, it should be feasible to grow epitaxial InAs/Al interfaces with ZnTe or CdSe as a tunnel barrier.

Here, we use DFT with machine-learned Hubbard U corrections^55^ to assess ZnTe and CdSe as prospective tunnel barriers for the InAs/Al interface. We conduct simulations of bulk ZnTe and CdSe, bilayer (110) interfaces of InAs/ZnTe, InAs/CdSe, CdSe/ZnTe, and (InAs,ZnTe,CdSe)/Al, as well as trilayer (110) interfaces of InAs/Al with a ZnTe or CdSe tunnel barrier of varying thickness. The choice of (110) interfaces is determined by the nanowire facets. For ZnTe, the DFT+U(BO) results are verified by comparison with published angle-resolved photoemission spectroscopy (ARPES) data.^56^ We note that despite the limitations of DFT for the description of superconductivity,^57^ DFT simulaitons can offer valuable insights into interface properties, including the band alignment and the penetration depth of metal-induced gap states (MIGS) into the semiconductor.^28^ We find that 16 atomic layers of either ZnTe or CdSe are sufficient to completely insulate the InAs from MIGS emanating from the Al. However, the two materials significantly differ in their band edge positions affecting their band alignment with InAs and Al. As a result, only ZnTe provides an effective barrier for electrons and therefore it would be the better candidate to pursue experimentally.

## Methods

### Computational Details

DFT calculations were conducted using the Vienna Ab Initio Simulation Package (VASP)^58^ with the projector augmented wave method (PAW).^59,60^ The generalized gradient approximation (GGA) of Perdew, Burke, and Ernzerhof (PBE)^61^ was employed to describe the exchange–correlation interactions among electrons with a Hubbard *U* correction.^62^ The *U* values were machine learned using Bayesian optimization (BO).^55^ Briefly, the BO objective function is formulated to reproduce as closely as possible the band structure obtained from the Heyd-Scuseria-Ernzerhof (HSE)^63,64^ hybrid functional. The reference HSE calculations were conducted using the experimental lattice parameters of 6.101 Å for ZnTe^33,34,65^ and 6.077 Å for CdSe.^38,66^ For ZnTe, a comparison to the results obtained with the lattice constant of InAs, 6.0584 Å, which was used for interface models, is provided in Figure S1 in the Supporting Information. We verified that using the lattice constant of InAs does not have an appreciable effect on the electronic properties of ZnTe (the lattice parameter of CdSe is sufficiently close to that of InAs that such a comparison was deemed unnecessary).

The hyperparameters of our BO implementation are the coefficients α_1_ and α_2_, which assign different weights to the band gap vs the band structure in the objective function, the number of valence and conduction bands used for the calculation of the objective function, *N*_b_, and the parameter κ that controls the balance between exploration and exploitation in the upper confidence bound acquisition function. The hyperparameters κ = 5, *N*_b_ = (5, 5), α_1_ = 0.25 and α_2_ = 0.75 were used here to find optimal *U* values for the Zn and Cd d-states, which dominate the top of the valence band and the bottom of the conduction band (see orbital decomposition provided in Figure S2 in the Supporting Information). The latter two parameters were chosen to assign more weight to the band shape rather than the band gap. *U* values of 9.4 and 8.3 eV were obtained for ZnTe and CdSe, respectively. Sensitivity analysis of the effect of changes in the *U* values on the resulting band structure is provided in Figures S3, S4 and Tables S1, S2 and BO convergence plots are provided in Figure S5 in the Supporting Information. For InAs, the *U* values of −0.5 and −7.5 eV were applied to the p-orbitals of In and As, respectively, following refs (55 and 67). It has been shown previously that with these parameters PBE+U(BO) produces a band structure in good agreement with ARPES for InAs.^67^ For structural relaxation at interfaces, the Tkatchenko-Scheffler (TS) method was used to account for dispersion interactions using the tags IVDW = 20 and LVDW_EWALD = True.^68^

Spin–orbit coupling (SOC) was used in all calculations and dipole corrections were applied to slab models.^69^ The tags used for convergence of calculations were BMIX = 3, AMIN = 0.01, ALGO = Fast, and EDIFF = 1 × 10^–5^. The kinetic energy cutoff was set to 400 eV for all bulk calculations and 350 eV for surface and interface slab models. A 7 × 7 × 7 *k*-point mesh was used for bulk calculations. For 1 × 1 slab models a *k*-point mesh of 7 × 7 × 1 was used for the self-consistent field (SCF) cycle and a *k*-point mesh of 13 × 13 × 1 was used for density of states (DOS) calculations. For supercell slab models (2 × 2 for the semiconductor and 3 × 3 for Al) a *k*-point mesh of 5 × 5 × 1 was used for the SCF cycle and a 9 × 9 × 1 *k*-point mesh was used for DOS calculations. Interface optimization calculations were conducted using a *k*-point grid of 3 × 3 × 1. The recommended POTCAR provided by VASP was used for all materials except In, for which POTCAR_In was used.

For slab calculations, a vacuum region of around 60 Å was added to prevent spurious interactions between periodic replicas. This amount of vacuum has been previously demonstrated to be well-converged.^70^ Semiconductor surfaces were passivated by pseudohydrogen atoms to avoid surface states from dangling bonds.^70^ The pseudohydrogen fractional charges utilized to passivate each atom were 1.25 for In and 0.75 for As in InAs, 1.5 for Zn and 0.5 for Te in ZnTe, 1.5 for Cd and 0.5 for Se in CdSe. Structural relaxation of the pseudohydrogen atoms was performed until the maximal force was below 0.001 eV/Å.

### Interface Model Construction

**Figure 1:**
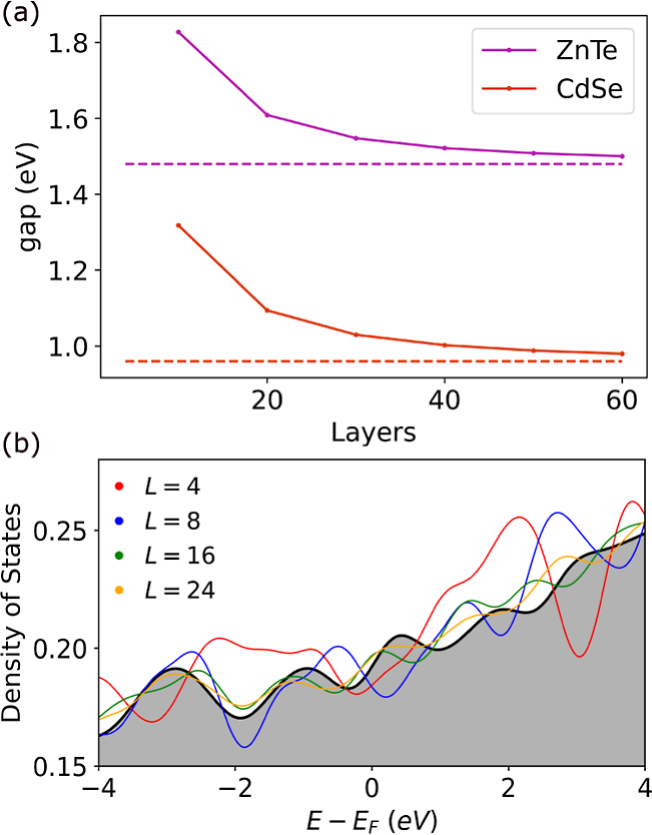
Thickness convergence: (a) the Γ-point band gap of ZnTe(110) and CdTe(110), using the InAs lattice constant, as a function of number of atomic-layers. Dashed lines denote the PBE+U(BO) bulk limit of 1.484 eV for ZnTe and 0.967 eV for CdSe. (b) Thickness convergence of Al(110): the local DOS for the middle layer of the slab is given as a function of number of atomic layers (L). The thick black line denotes the PBE+U(BO) bulk density of states. Gaussian broadening with σ = 0.3 was applied to the data.

All interface models were constructed with a (110) orientation using the experimental InAs lattice constant value of 6.0584 Å,^37,71^ based on the assumption that the epitaxial films would conform to the substrate. When constructing slab models, it is necessary to converge the number of layers to avoid quantum size effects and approach the bulk properties.^70^ For InAs it has been shown previously that 50 atomic layers are sufficiently converged.^67^Figure 1a shows the Γ-point band gap obtained with PBE+U(BO) as a function of the number of atomic layers for ZnTe(110) and CdSe(110) slabs. The band gap is considered converged when the band gap changes by less than 5 × 10^–2^ eV when more layers are added. ZnTe and CdSe are deemed converged with 40 atomic layers. For metal slabs, convergence is determined based on the DOS.^70^Figure 1b shows the local DOS at the center of a slab with a varying number of Al layers, compared to the bulk DOS (obtained for a unit cell with the same orientation), calculated using the experimental lattice parameter of 4.0389 Å. With 4 atomic layers the DOS deviates very significantly from the bulk DOS. With 8 layers the DOS at the center of the slab starts to resemble the bulk DOS, and with 16 layers the DOS is close to converged relative to the bulk DOS, in particular around the Fermi level. We note that even with 4 layers the Al slab is already metallic and the fluctuations in the DOS around the Fermi level with an increasing number of layers are small. In this sense, metals are less sensitive to thickness convergence than semiconductors, semimetals,^28^ and topological materials.^72^

**Figure 2:**
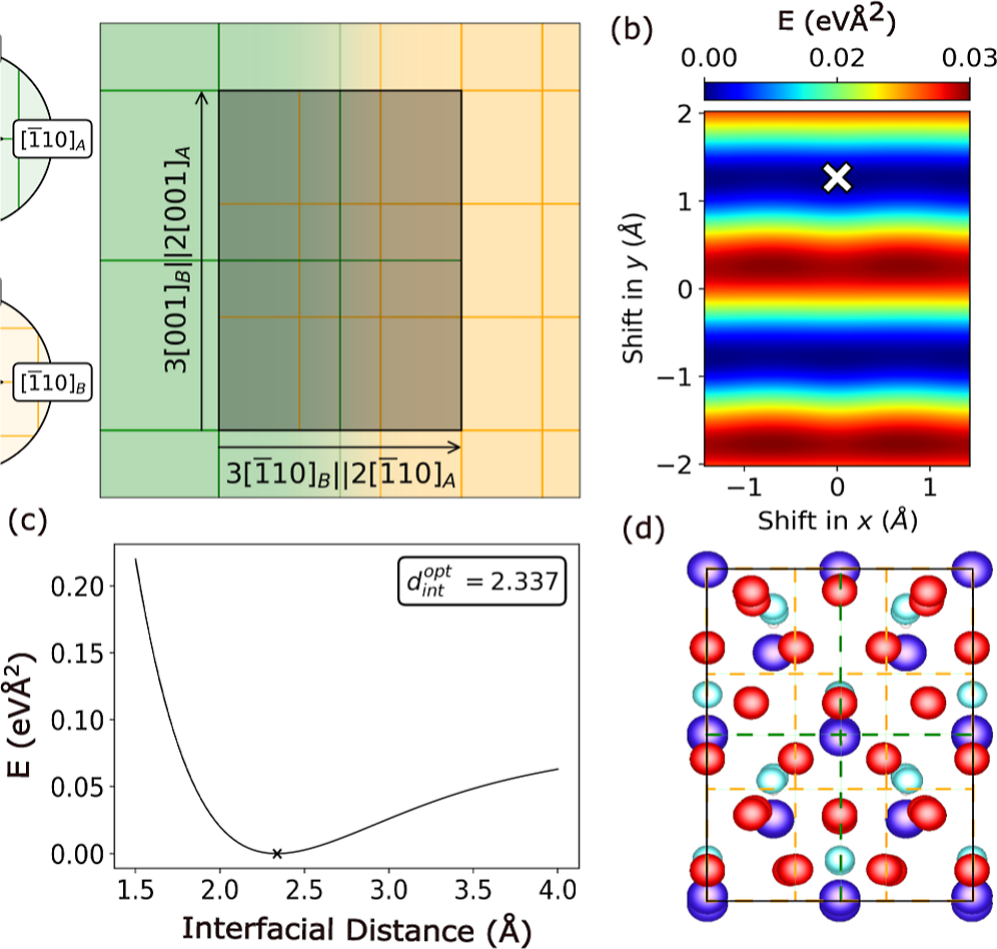
Interface construction for the InAs/Al interface. (a) domain-matched supercells constructed from a 2 × 2 supercell of InAs (A, green) and a 3 × 3 supercell of Al (B, orange). The arrows indicate the surface parallel crystallographic directions along **x** and **y** of the film and substrate. (b) Potential energy change as a function of the registry between the film and substrate with the minimum set to zero. Only one symmetry-unique tile is shown with the location of the minimum indicated by a cross. (c) Binding energy curve as a function of interfacial distance. (d) Top-view of the structure of the optimized interface configuration with Al atoms colored in red, In atoms colored in purple, and As atoms colored in light blue.

Interface models were constructed using the Ogre code.^70,73^ For semiconductor/Al interfaces, lattice matching was performed, resulting in a domain-matched interface comprising a 2 × 2 supercell of the semiconductor and a 3 × 3 supercell of Al with a mismatch of less than 0.1%, as shown in Figure 2a. To determine the optimal position of the film on top of the substrate and the bonding configuration across the interface, a potential energy surface (PES) was calculated by shifting the film on top of the substrate parallel to the plane of the interface. This was done at an interfacial distance of the average interlayer distance of the film and substrate structures, given by , which resulted in 1.784 Å for the semiconductor/Al interfaces and 2.142 Å for the semiconductor/semiconductor interfaces. Figure 2b shows an example for InAs/Al and plots for the remaining bilayer interfaces are provided in Figures S7 and S8 in the Supporting Information. The most stable configuration of the InAs/(ZnTe,CdSe) interfaces is found to be with In–Te,Se and As–Zn,Cd bonding, consistent with experimental observations.^36,74^ The ZnTe/CdSe interface has Zn–Se and Te–Cd bonding, also consistent with experiment.^75^ For the most stable bonding configuration, a binding energy curve was calculated perpendicular to the plane to find the optimal interfacial distance. A value of 2.337 Å was obtained for the InAs/Al interface, as shown in Figure 2c. The optimal interfacial distances found for the remaining interfaces are 2.465 Å for ZnTe/Al, 2.5 Å for CdSe/Al, 2.11 Å for InAs/ZnTe, 2.1875 Å for InAs/CdSe, and 2.163 Å for ZnTe/CdSe. For computational efficiency, the PES and binding energy curves were calculated without SOC, using 4 atomic layers of each material.

Finally, a few layers near the interface were relaxed, with SOC applied, using a slab with 8 atomic layers of each material. For semiconductor/semiconductor interfaces, two atomic layers were relaxed on either side, and for semiconductor/Al interfaces four atomic layers were relaxed on either side. A representative example of the relaxed InAs/Al interface is shown in Figure 2d. The relaxed geometries of the bilayer interfaces were used to construct trilayer interfaces. For trilayer interfaces with fewer than 8 barrier layers, the entire interface region was relaxed. The bilayer semiconductor interfaces, which do not necessitate supercells in the plane of the interface, comprised 40 atomic layers of each material. Owing to the increased computational cost of the supercells used to construct the interfaces with Al, the bilayer interfaces contained 30 layers of the semiconductor and 16 layers of Al. The trilayer interface models comprised 20 layers of InAs, 8 layers of Al and up to 24 atomic layers of ZnTe or CdSe, amounting to a total slab thickness of around 9 nm (not including vacuum) with a barrier of up to 5 nm. The smaller number of semiconductor layers in these interfaces results in a somewhat wider gap, owing to the quantum size effect (see Figure 1 and ref (67)). Ideal abrupt interfaces were considered, with no intermixing.

## Results and Discussion

### Bulk ZnTe and CdSe

**Figure 3:**
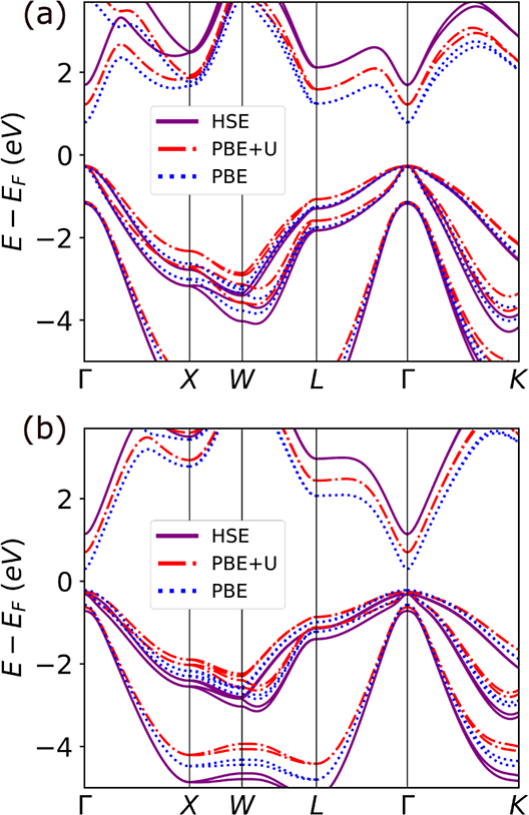
Comparison between the band structures obtained using PBE, HSE, and PBE+U(BO) for (a) ZnTe and (b) CdSe.

Figure 3 shows the bulk band structures of ZnTe and CdSe obtained with different exchange–correlation functionals. The magnitude of the band gap is the main difference between the results of different functionals. For both compounds, the transition metal d states contribute significantly to the top of the valence band and the bottom of the conduction band at the Γ point, as shown in Figure S2 in the Supporting Information. The PBE functional significantly underestimates the band gap of both compounds, yielding 1.07 eV for ZnTe compared to the experimental values of 2.26 at ambient temperature^47,76^ and 2.38 eV at 0 K.^65,77,78^ For CdSe, PBE yields a band gap of 0.51 eV compared to the experimental value of 1.75 eV.^33,35,66^ The band gap underestimation of (semi)local functionals has been attributed to the self-interaction error (SIE), a spurious repulsion of an electron from its own charge density, and to the absence of the expected discontinuity of the derivative of the total energy with respect to changes in the number of electrons.^79^ Highly localized states, such as d orbitals, are typically affected more strongly by SIE.

Hybrid functionals, which include a fraction of exact (Fock) exchange, mitigate the effect of SIE and yield band gaps in better agreement with experiment.^80,81^ The HSE functional yields improved band gaps of 1.86 eV for ZnTe and 1.46 eV for CdSe. However, the computational cost of hybrid functionals is too high for simulations of large interface systems, such as the InAs/ZnTe,CdSe/Al trilayer systems studied here. The DFT+U approach, whereby a Hubbard *U* correction is added to certain atomic orbitals, provides a good balance between accuracy and computational cost.^80,81^*U* parameters determined by Bayesian optimization^55^ are applied to the d-states of Zn and Cd, as described in the Computational Details section. The PBE+U(BO) band gaps of 1.48 eV for ZnTe and 0.96 eV for CdSe are a significant improvement over PBE but still lower than the HSE and experimental values. Indeed, for ZnTe, it has been reported previously that even with very large *U* values the band gap is still considerably narrower than the experimental value.^82^

### Comparison to ARPES for ZnTe

**Figure 4:**
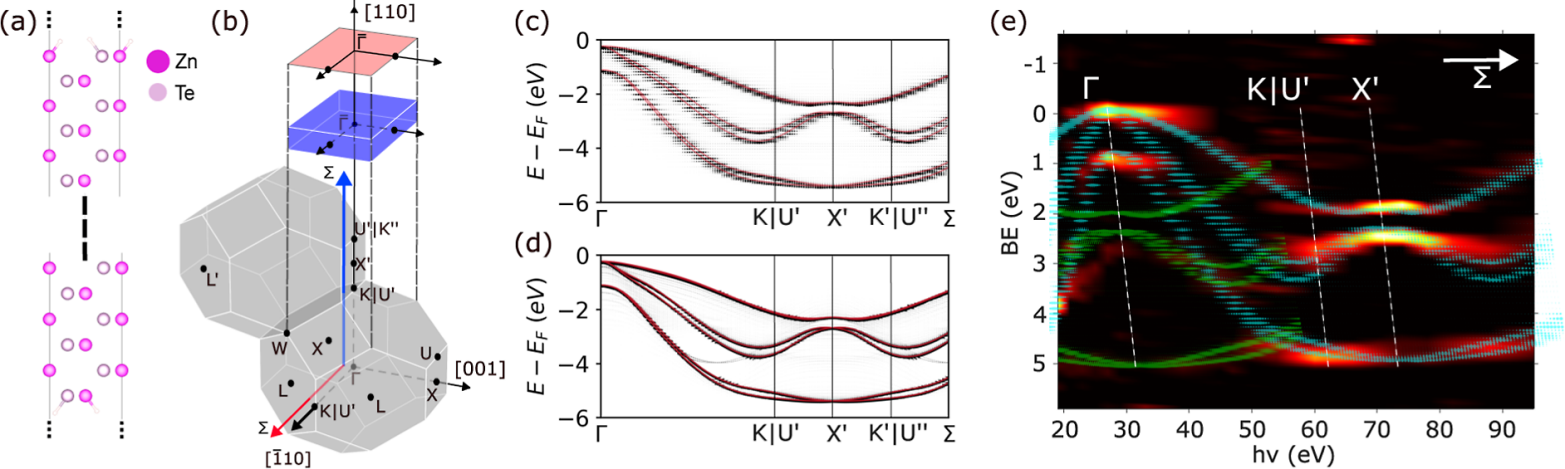
Electronic structure of ZnTe: (a) illustration of the 50 layer ZnTe slab (with vacuum). (b) Illustration of the Brillouin zone (BZ) of bulk FCC (gray), the (110) 2D surface BZ (red), and the BZ of a slab model with a finite thickness (blue). The surface parallel and surface perpendicular Γ – *K*|*U* – *X* paths are indicated by the red and blue arrows, respectively. Bulk-unfolded band structures along the (c) surface-perpendicular and (d) surface-parallel paths. The corresponding bands of bulk ZnTe are shown in red in both panels. (e) Bulk-unfolded band structure of the surface perpendicular Γ – *K*|*U* – *X* path (cyan) compared to the ARPES data adapted with permission from “Angle-resolved photoelectron spectroscopy study of the surface electronic structure of ZnTe(110)” by Qu et al., Phys. Rev. B, 43, 9843 (1991); Copyright (1991) by the American Physical Society.^56^ The bands colored in green are shifted by a |**ΓX**^′^| lattice vector to reproduce the Umklapp bands in the ARPES.

The most powerful experimental tool for verifying the validity of electronic structure models is ARPES. This technique is able to resolve the electronic structure in three-dimensional momentum, **k**, space. The surface-parallel momentum, **k**_*xy*_, is varied through the photoelectron emission angle (ϑ), whereas the surface-perpendicular component, *k*_*z*_, is varied through the photon energy (*hv*) (see, for example refs (83 and 84)). The PBE+U(BO) results for ZnTe are verified by comparison with the ARPES data for ZnTe(110) reported in ref (56). Often, ARPES experiments are compared to bulk band structure calculations. However, bulk band structures cannot capture surface and interface phenomena, which require technically more demanding slab calculations. Here, the ZnTe(110) surface is simulated using a slab model, shown in Figure 4a. The supercell extends over 50 unit layers in the surface-perpendicular direction and includes an additional vacuum region. Such supercell models produce band structures that appear as a dense interwoven manifold of bands as a function of **k**_*xy*_, as shown in Figure S6 in the Supporting Information. Each band within this manifold is characterized by a certain, albeit smeared, value of *k*_*z*_ in the Fourier expansion of the corresponding wave function.^85^ The relation between such band structure calculations and ARPES spectra, which is far from obvious, can be recovered by using so-called “band unfolding”. In this approach, the band structure generated from supercell calculations is projected, or unfolded, onto the appropriate smaller unit cell.^86^ The band manifolds in the small Brillouin zone of the supercell then split into the separate bands in the larger Brillouin zone of the smaller unit cell, untangling the interwoven manifold of bands. This can help resolve e.g., the contributions of surface states vs the bulk bands of the material.^28,67^

In the bulk Brillouin zone, the surface-perpendicular Γ – *K*|*U*′ – *X*′ direction is symmetry equivalent to the surface-parallel Γ – *K*|*U*′ – *X*′ directions, as indicated in Figure 4b by the blue and red arrows, respectively. However, the finite length of the slab in the surface-perpendicular direction breaks this symmetry and produces a Brillouin zone that is very thin in the surface-perpendicular direction. This manifests in the differences between the unfolded band structures along the surface-parallel vs the surface-perpendicular paths. Along the surface-parallel path, shown in Figure 4c, full periodicity is retained, producing a bulk-like band structure. Confinement in the surface-perpendicular direction (between the vacuum regions of the supercell) leads to discretization of *k*_*z*_, as well as its broadening, owing to the Heisenberg uncertainty principle. This manifests as ladder-like horizontal stripes in the unfolded band structure, as seen in Figure 4d (additional plots of the dependence of the unfolded surface perpendicular band structure on the slab thickness are provided in Figure S6 the Supporting Information, further confirming the quantum-confinement origin of the discrete broadened energy levels).

In Figure 4e, the unfolded band structure along the surface-perpendicular path Γ – *K*|*U*′ – *X*′ is compared to the ARPES data reported in ref (56). Figure 1 therein presents the ARPES spectra collected at normal emission (corresponding to **k**_*xy*_ = 0) under variation of *hv* over the range 19–95 eV, whereby **k**_*z*_ is varied along the out-of-plane direction Γ – *K*|*U*′ – *X* (the Σ line) of the bulk Brillouin zone of ZnTe. Figure 4e shows the spectra digitized from the original figure and plotted in the binding energy (*E*_*b*_) vs *hv* coordinates as the negative second derivative of the ARPES intensity (−d^2^*I*/d*E*_*b*_^2^) represented by the heat color scale. The *E*_*b*_ scale here is referenced to the valence band maximum (VBM). The APRES data without the computed band structure is provided in Figure S7 in the Supporting Information. For the comparison to ARPES, the *k*_*z*_ coordinates of calculated points were converted to *hv* coordinates, assuming free-electron final states, by: *hv* = (ℏ^2^/2*m*_0_) × (*k*_*z*_ + *G*_*z*_)^2^ – *V*_000_ + *E*_*b*_. Here, *m*_0_ is the free-electron mass, *G*_*z*_ runs through surface-perpendicular bulk reciprocal vectors, and the inner potential *V*_000_ is chosen as 5 eV.^56^

The unfolded band structure is in good agreement with the ARPES data. The discrete, broadened energy levels hardly affect the comparison because their energy separation is less than the energy broadening of the experimental dispersions due to the finite hole lifetime and the experimental energy resolution. The broadening effects in the experiment also explain why the splitting of the calculated bands around the *K*|U′ point is not resolved. In the lower left region of panel (e) there are ARPES peaks that do not correspond to any of the DFT bands. Their dispersion can be described, however, with the same theoretical valence bands but with the final states shifted by a certain Δ*k*_*z*_ from the free-electron ones. The theoretical dispersion in the (*hv*, *E*_*b*_) coordinates then becomes . Therefore, in this *hv* range, the ARPES final state incorporates two bands, one having the free-electron *k*_*z*_ and another having *k*_*z*_ + Δ*k*_*z*_ shifted from the free-electron dispersion, with the ARPES intensity distribution between these bands defined by the energy- and ***k***-dependent photoemission matrix elements. In our case, we find Δ*k*_*z*_ ≈ 1.0371 Å^–1^, equal to |ΓX^′^|. Conventionally, such final-state bands are called Umklapp bands, implying an Umklapp scattering process of the photoelectrons,^56^ although the corresponding momentum transfer is not necessarily an integer reciprocal-lattice vector. A rigorous description of such multiband final states based on the one-step theory of photoemission, where they are treated as the time-reversed low-energy electron diffraction (LEED) states, can be found in refs (87–89) and the references therein.

### Semiconductor Interfaces

Determining the band alignment in semiconductor/semiconductor heterojunctions is important for understanding and improving the performance of semiconductor devices such as lasers, photovoltaic cells, and transistors.^90^ The band edge positions of the two separate materials may be considered as a zero-order approximation for the band alignment at an interface. The bonding configuration and orientation between the two materials and the presence of an interface dipole, as well as the presence of defects can lead to significant differences.^36,47,91−93^ This may explain some of the variation between experimentally reported values.

Simulations can help interpret experiments, as well as predict the band alignment produced by different combinations of semiconductors.^94^ The underestimation of band gaps by (semi)local DFT functionals can lead to errors in the computed band alignment. In principle, the interface band alignment could be calculated using the GW approximation within the framework of many-body perturbation theory. However, owing to the high computational cost, simulations of sufficiently large interface models with enough layers to approach the bulk band gaps are not currently feasible using GW or even hybrid DFT functionals. To circumnavigate this problem, corrections based on the shift of the electrostatic potential can be applied to the bulk band edge positions. The electrostatic potential lineup is relatively insensitive to the method used and converges fast with the number of layers.^95,96^ Therefore, the electrostatic potential correction can be calculated using a more computationally efficient method (e.g., semilocal DFT), while the bulk band structure is calculated using a more accurate and expensive method (e.g., GW or hybrid DFT).^39,95−97^ The electrostatic potential correction may be performed for separate surface slabs of each material or for periodic heterostructures with a relatively small number of layers of each material.^98^ Additional corrections may be performed at the semilocal DFT level e.g., to account for the effect of interface strain,^95^ and the formation of a dipole in polar interfaces^91,93^ Thus, expensive calculations of large interface models can be avoided.

A limitation of approaches that avoid direct modeling of the interface is that they cannot describe phenomena, such as band bending and the penetration of states from one material into the gap of the other in the vicinity of the interface. Modeling these phenomena requires a sufficient number of layers to span their length-scale. We also note that it has been shown that structural relaxation at the interface can have a significant effect on the band alignment,^91^ and this is indeed the case for the interfaces studied here, as discussed below. Therefore, if periodic interface models with a small number of layers are used to calculate electrostatic potential corrections, care should be taken to include and relax a sufficient number of layers around the interface. In our case, the main drawback of approaches that avoid direct simulations of large interface models is that they cannot be used to assess the effectiveness of a tunnel barrier, which is the goal of this work.

**Figure 5:**
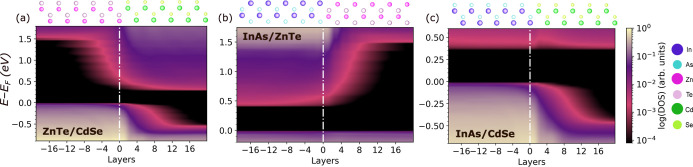
Electronic structure of semiconductor–semiconductor bilayer interfaces: density of states as a function of position across the (a) ZnTe/CdSe, (b) InAs/ZnTe and (c) InAs/CdSe interfaces. The atomic layers are numbered based on distance from the interface, which is located at zero. Illustrations of the interface structures are also shown.

Figure 5 shows the DOS as a function of position across the three semiconductor interfaces. Element-resolved band structures are shown in Figure S11 in the Supporting Information. ZnTe and CdSe differ significantly in their band edge positions with respect to each other and to InAs. Panel (a) shows that ZnTe and CdSe form a type-II interface with staggered band gaps. Close to the interface, there is significant penetration of states from the bands of one material into the gap of the other. Therefore, the band offsets are extracted from the asymptotic band edge positions. We find a valence band offset (VBO) of 0.5 eV and a conduction band offset (CBO) of 1.2 eV. These values are within the range of other first-principles studies, which reported VBOs of 0.5–0.9 eV and CBOs of 1.0–1.4 eV.^39,91,94,99^ Prior to relaxation, we obtained a type-II band alignment, but with a VBO of 0.15 eV and a CBO of 0.85 eV, as shown in Figure S10 in the Supporting Information.

All the experimental band offset data available are for ZnTe/CdSe interfaces with a (001) orientation. Typically, only the VBO is measured directly and the CBO is deduced based on the bulk band gaps of the two materials. Yu et al. have reported a VBO of 0.64 ± 0.07 eV using X-ray photoelectron spectroscopy (XPS).^43^ Mourad et al.^42^ have reported a VBO of 0.75 ± 0.01 eV, based on photoluminescence (PL) measurements. Gleim et al. and Sedova et al. conducted experiments on trilayer junctions of CdSe/ZnTe/InAs.^36,47^ Gleim et al. conducted photoelectron spectroscopy (PES) measurements for both thin (three monolayer) and thick films of ZnTe. They reported a VBO of 0.6 ± 0.1 eV. Sedova et al. conducted their experiments only with a three monolayer film of ZnTe and reported a significantly different VBO of 0.94 eV. Depending on what VBO was measured and what values were used for the band gaps of both materials, the CBO has been estimated as 1.09,^47^ 1.22,^43^ and 1.54 eV.^36^ Despite the band gap underestimation by PBE+U(BO) the band offsets are in good agreement with experimental values.

Panel (b) shows that InAs and ZnTe form a type-I interface with the valence band maximum aligned to give a VBO of zero and a CBO of 1.1 eV. States penetrate from the InAs conduction band into the band gap of the ZnTe and decay within about 14 atomic layers from the interface. The band offsets obtained here are close to the VBO of 0.2 eV and CBO of 1.1 eV computed in ref (39). We note, however, that structural relaxation, which was not performed in ref (39), significantly affects the resulting band alignment of the InAs/ZnTe interface. Prior to relaxation, we obtained a VBO of 0.35 eV and CBO of 0.6 eV, as shown in Figure S10 in the Supporting Information. Experiments have been conducted only for InAs/ZnTe interfaces with a (001) orientation. VBO values of 0.2 to 0.3 eV have been reported.^36,47^ The presence of a dipole at the (001) interface may cause some differences in the band offsets compared to the nonpolar (110) orientation.

Pairing InAs with CdSe leads to an opposite band alignment to ZnTe, as shown in Panel (c). The InAs/CdSe interface is also type-I, but with the bottom of the InAs and CdSe conduction bands aligning to produce a CBO of zero. We find a VBO of 0.425 eV. States penetrate from the valence band of the InAs into the gap of the CdSe and decay within 14 atomic layers. In this case, geometry relaxation qualitatively changed the band alignment. Before relaxation, a type-II band alignment was found with a CBO of 0.1 and a VBO of 0.6 eV, as shown in Figure S10 in the Supporting Information. Reference (39) has reported a type I interface with a small CBO of 0.16 eV, but a significantly lager VBO of 1.25 eV. Two experimental values have been reported for the VBO for (001) InAs/CdSe interfaces. Reference (47) has reported VBO of 0.86 ± 0.15 eV, whereas ref (36) has reported a value of 1.05 eV (we note that ref (36) appears to systematically report higher VBO values than ref (47)). Our results are qualitatively consistent with the values reported in other computational and experimental studies. The somewhat larger differences for the CdSe/InAs interface may be attributed to the underestimation of the band gap of CdSe by PBE+U(BO) and the different interface orientation.

### Semiconductor/Al Interfaces

The band alignment at semiconductor/metal interfaces is a key parameter for device performance because it fundamentally affects the contact resistance and therefore the charge transport through metal contacts.^100,101^ Depending on the semiconductor band edge positions with respect to the metal work function, either an Ohmic contact or a potential step, known as a Schottky barrier, may form.^102,103^ At low temperatures, Ohmic contact interfaces may accommodate proximity-induced superconductivity in the semiconductor, while Schottky barrier interfaces would likely lead to a suppressed proximity effect. It has been established, both theoretically and experimentally, that the local properties of the interface, including the crystal lattice orientation, the bonding configuration, presence of defects, charge redistribution, and dipole formation, may further affect the band alignment and Schottky barrier height (SBH).^101,104−107^ First-principles simulations can help interpret experiments and predict the band alignment at metal/semiconductor interfaces. To this end, the electrostatic potential alignment method, described in detail in the Supporting Information, is often used.^96,108,109^ The idea is similar to the method described above for determining the band alignment at semiconductor interfaces. To avoid expensive calculations of large slab models, and to compensate for errors caused by the underestimation of band gaps by semilocal functionals, the SBH is evaluated by referencing the average electrostatic potential difference between the semiconductor and metal, calculated using a smaller slab model, to calibrate bulk values of the Fermi energy and band edges. A detailed description of the procedure is provided in eqs 1–6 in the Supporting Information. Similar to the band alignment at semiconductor interfaces discussed above, the SBH results can be sensitive to geometry relaxation in the interface region.^106,108^ As explained above, we are ultimately interested in studying the spatial evolution of the electronic structure as a function of position across the interface. To describe local effects, such as the presence of MIGS, which manifest as a finite density of states in the gap of the semiconductor due to the penetration of exponentially decaying metallic Bloch states,^110−112^ fully converged slab models are used. Below, the band alignment and SBH values are extracted from the local DOS of large slab models. For completeness, we also provide values calculated using the potential alignment method. A detailed description of these calculations is provided in Figure S14 and Tables S3 and S4 in the Supporting Information. For all systems studied here, the two methods yield values within ∼0.1 eV of each other.

**Figure 6:**
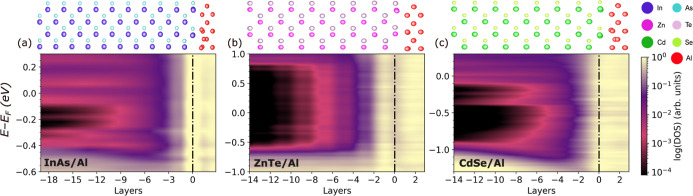
Electronic structure of semiconductor/Al bilayer interfaces: Density of states in the (a) InAs/Al, (b) ZnTe/Al and (c) CdSe/Al interfaces as a function of position. The atomic layers are numbered based on distance from the interface, which is located at zero. The structure of each interface is illustrated on top.

Figure 6 shows the DOS as a function of position across interfaces of the three semiconductors with Al. In all three interfaces there is a significant presence of MIGS, which decay gradually with the distance from the interface, as also seen in the local DOS plots provided in Figure S12 in the Supporting Information. In ZnTe, the MIGS decay more rapidly than in the CdSe and InAs, vanishing completely within 14 atomic layers. In the InAs and CdSe some MIGS persist about 18 layers (∼4 nm) deep into the semiconductor. Deep MIGS are also seen in the structurally similar GaSb/Al interface, shown in Figure S13 in the Supporting Information. Based on Figure 6a, in the InAs/Al interface there is downward band bending with the conduction band minimum (CBM) lying ∼0.1 eV below the Fermi level. SBH calculations using the potential alignment method yield a negative value of −0.05 eV, which also indicates an Ohmic contact. These results are consistent with experimental observations in ref (113), which measured a downward band bending of around 0.3 eV for InAs(100)/Al interfaces, and with the calculations reported in ref (114) for InAs(100)/Al(111). Figure 6b shows that in the ZnTe/Al interface there is no band bending and the Fermi level is located in the ZnTe gap with a SBH of ∼0.8 eV. The potential alignment method yields a SBH of 0.9 eV. These results are consistent with reports of a Schottky barrier of 0.9 eV for ZnTe(111)^50^ and ZnTe(110)^51,52^ interfaces with Al. Figure 6c shows that in the CdSe/Al interface there is downward band bending, similar to InAs/Al, with the CBM positioned ∼0.1 eV below the Fermi level. The potential alignment method yields a value of 0.01 eV. Our results are consistent with experimental reports of Ohmic contacts for CdSe(101̅0) interfaced with Al.^53,115,116^

### Trilayer Interfaces

**Figure 7:**
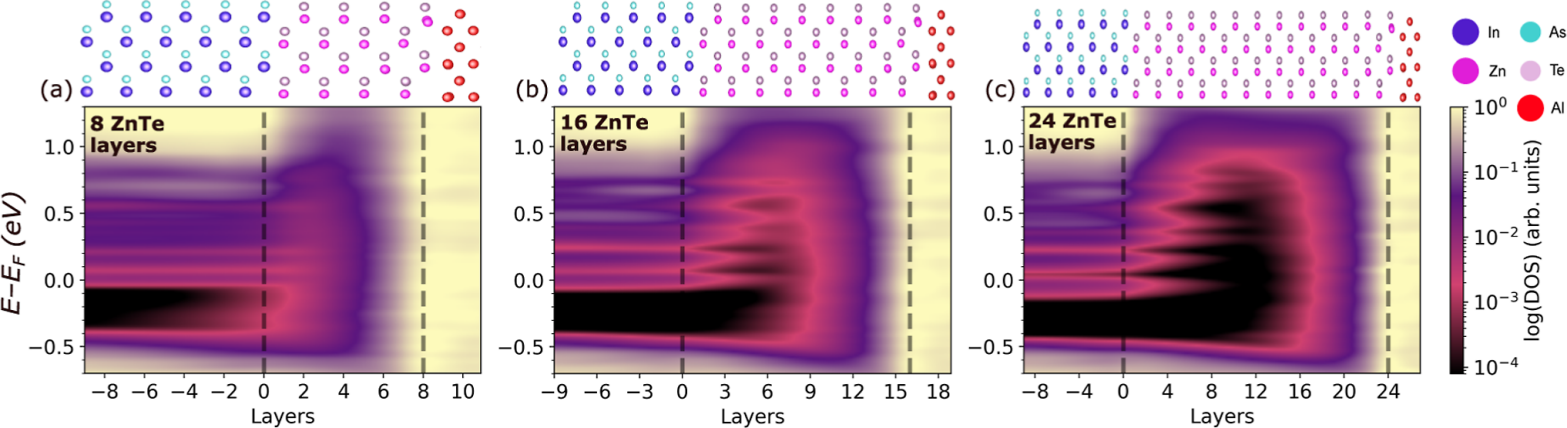
Electronic structure of InAs/ZnTe/Al trilayer interfaces: Density of states as a function of distance from the interface for (a) 8, (b) 16 and (c) 24 atomic layers of ZnTe. The atomic layers are numbered based on distance from the InAs interface, which is located at zero. The location of the InAs/ZnTe and ZnTe/Al interfaces is indicated by dashed lines. The structure of each interface is illustrated on top.

**Figure 8:**
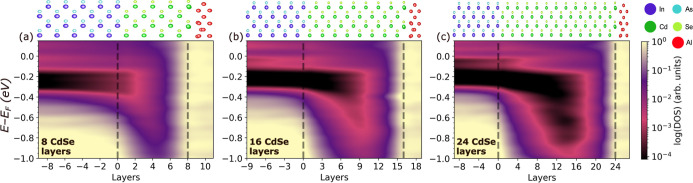
Electronic structure of InAs/CdSe/Al trilayer interfaces: Density of states as a function of distance from the interface for (a) 8, (b) 16 and (c) 24 atomic layers of CdSe. The atomic layers are numbered based on distance from the InAs interface, which is located at zero. The location of the InAs/CdSe and CdSe/Al interfaces is indicated by dashed lines. The structure of each interface is illustrated on top.

Figures 7 and 8 show the DOS as a function of position across trilayer interfaces, in which a ZnTe or CdSe tunnel barrier with varying thickness is inserted between InAs and Al. Results for Interfaces with 8, 16, and 24 layers of the tunnel barrier are shown here and additional results are provided in Figures S18 and S19 in the Supporting Information. In all cases, regardless of the barrier material and thickness, there is downward band bending and the Fermi level remains in the InAs conduction band. The tunnel barrier does not unpin the InAs Fermi level. With 8 atomic layers of either material, the MIGS from the Al penetrate through the tunnel barrier into the InAs. As the barrier thickness increases, the InAs becomes increasingly insulated from MIGS until with 16 barrier layers the InAs and Al are completely decoupled from each other. This is also evident from the local DOS in the second InAs layer from the interface, provided in Figure S15 the Supporting Information.

The evolution of the ZnTe and CdSe band gaps with the number of layers is similar to that of the CdTe tunnel barrier at the InSb/α-Sn interface, reported previously.^28^ On the one hand, for a thin layer of the barrier material the band gap is expected to be significantly larger than the bulk value because of the quantum size effect (see Figure 1a). On the other hand, there are MIGS penetrating into the gap of the barrier from the Al side and InAs states penetrating from the other side (states from the InAs conduction band penetrate into the ZnTe and states from the InAs valence band penetrate into the CdSe, as seen in Figure 5b,c). For 8 atomic layers of barrier material the MIGS from the Al completely fill the ZnTe/CdSe band gap. With 16 atomic layers of barrier material the gap of the barrier remains smaller than its bulk value. For ZnTe the band gap reaches a maximum of 1.1 eV at a distance of 8 atomic layers from the Al interface. For CdSe the band gap reaches a maximum of 0.5 eV at a distance of 8 atomic layers from the Al interface. With 24 barrier layers the gap of the barrier approaches the expected value of 1.58 eV for ZnTe and 1.06 eV for CdSe obtained for a slab of the same thickness (see Figure 1a). For ZnTe the band gap reaches a maximum of 1.35 eV at a distance of 12 atomic layers from the Al interface. For CdSe the band gap reaches a maximum of 0.8 eV at a distance of 12 atomic layers from the Al interface. Additional local DOS plots showing the evolution of the barrier band gap are provided in Figures S16 and S17 in the Supporting Information.

Although ZnTe and CdSe exhibit similar characteristics in terms of the penetration of MIGS and the band gap evolution with barrier thickness, they significantly differ in their band edge positions. For the ZnTe barrier, the valence band maximum is aligned with that of the InAs, similar to the bilayer interface (Figure 5b). Once the ZnTe barrier becomes thick enough to develop a band gap (Figure 7c) the Fermi level is positioned in the gap, similar to the bilayer interface with Al (Figure 6b). This means that ZnTe forms an effective barrier for electrons. Owing to the slight downward band bending, a small barrier for holes is also present in Figure 7c. For the CdSe barrier, the conduction band minimum is aligned with that of the InAs, similar to the bilayer interface (Figure 5c). Regardless of the barrier thickness, the Fermi level is positioned in the CdSe conduction band, similar to the bilayer interface with Al (Figure 6c). As a result, CdSe forms an effective barrier for holes but no barrier for electrons. In InAs-based Majorana devices, there is only electron transport because of Fermi level pinning in the conduction band at the interface. Therefore, of the two materials considered here, ZnTe is the better candidate for a tunnel barrier. CdSe may still serve as a spacer layer and result in interface scattering providing an effective barrier, but it would be difficult to control the transmission through this barrier. Similar to our previous study of CdTe as a prospective barrier material for the InSb/α-Sn interface,^28^ we consider the presence of MIGS as an indicator for coupling between the Al and InAs. Therefore, we suggest the thickness range between 6 and 18 atomic layers for experimental exploration.

## Conclusion

In summary, we have used DFT with Hubbard *U* corrections machine-learned by Bayesian optimization to study ZnTe and CdSe as prospective tunnel barrier materials for the InAs/Al interface. For ZnTe good agreement is obtained between the PBE+U(BO) band structure and ARPES experiments, including reproducing the observed Umklapp states. PBE+U(BO) provides a balance between accuracy and computational cost that enables calculations of very large interface models.

We then used PBE+U(BO) to calculate the electronic structure of bilayer semiconductor/semiconductor and semiconductor/Al interfaces. For the semiconductor interfaces, the type of band alignment produced by PBE+U(BO) is qualitatively correct and the band offsets are in reasonable agreement with experiments and other computational studies. For the semiconductor/Al interfaces, we find that MIGS penetrate into the semiconductor and decay within 14–18 atomic layers (∼4 nm). InAs and CdSe are found to exhibit downward band bending and an Ohmic contact at the interface, whereas for ZnTe the Fermi level is positioned in the band gap, forming a Schottky barrier. For all three semiconductor/Al interfaces, our results are in agreement with experimental observations. PBE+U(BO) thus provides a correct description of the band alignment at semiconductor/semiconductor and semiconductor/metal interfaces.

Finally, we considered trilayer interfaces with a varying number of atomic layers of the ZnTe or CdSe tunnel barrier between the InAs and Al. We find that the band gap of the barrier evolves as a function of distance from the InAs and Al interfaces in a similar manner to the Insb/CdTe/α-Sn interface we had studied previously.^28^ Contrary to the expectation that the band gap of a very thin barrier would be wider than the bulk band gap because of the quantum size effect, in fact, it is narrowed owing to evanescent states penetrating from both the Al and InAs directions. Only with 24 atomic layers of either material the barrier band gap approaches the expected value about halfway between the Al and InAs. With 8 atomic layers of either ZnTe or CdSe, MIGS emanating from the Al penetrate through the entire width of the barrier into the InAs. Sixteen layers of either material are sufficient to insulate the InAs from MIGS, which we interpret to mean that the InAs is effectively decoupled from the Al.

Although ZnTe and CdSe behave similarly with respect to the barrier thickness, the two materials markedly differ in their band edge positions. ZnTe provides an effective barrier for electrons, whereas CdSe only provides a barrier for holes. In devices based on InAs, holes cannot be utilized because of Fermi level pinning in the conduction band at the interface. As a result, transport is based only on electrons. Therefore, of the two materials considered here, only ZnTe could serve as an effective tunnel barrier in InAs-based Majorana devices (CdSe may still serve as a spacer layer).

We had previously postulated that in Majorana experiments a barrier thick enough to completely insulate the semiconductor from the superconductor might also nearly eliminate transport.^28^ We assume that the relevant regime for tuning the coupling at the interface would be in the thickness range where some MIGS are still present. Hence, based on the results presented here, we would suggest conducting a systematic series of experiments in which the thickness of a ZnTe tunnel barrier is varied between 6 and 16 atomic layers and the induced superconducting gap in the InAs is measured.

In the future, combining DFT with Bogoliubov-de Gennes theory could help establish a connection between parameters of the interface and proximity-induced superconductivity.^117,118^ Careful experimentation guided by theory could advance the design of devices with optimal interface configurations for the emergence of MZMs. More broadly, metal–semiconductor interfaces are ubiquitous in many types of devices. The results presented here demonstrate the prospects of DFT simulations as a powerful tool for optimizing interface configurations for semiconductor-based devices in general, including multilayer stacks comprising multiple materials and interfaces.

## Data Availability

VASP input files, raw data, data processing scripts and plotting scripts are available on Zenodo 10.5281/zenodo.13901100. An updated version of the BO code for determining the Hubbard U parameter, “BayesianOpt4dftu” is available at: https://github.com/caizefeng/BayesianOpt4dftu. The VaspVis package,^70^ used to make band structure and density of states plots is available on GitHub at: https://github.com/DerekDardzinski/vaspvis or from The Python Package Index (PyPI) via the command: *pip install vaspvis*.

## Supporting Information Available

Effect of lattice parameter on the band structure of ZnTe; Orbital decomposition of the band structures of ZnTe and CdSe obtained with different DFT functionals; Sensitivity analysis and convergence of PBE+U(BO); Effect of slab thickness on unfolded surface-parallel and surface-perpendicular band structures of ZnTe; Surface matching results for bilayer interfaces; Effect of relaxation on the band alignment at semiconductor interfaces; Element-resolved band structures of semiconductor interfaces; Local DOS for semiconductor/Al interfaces; DOS as a function of position and local DOS for the GaSb/Al interface; SBH calculations using the electrostatic potential alignment method; Local DOS for trilayer interfaces; DOS as a function of position for trilayer interfaces with additional barrier thickness values (PDF)
