# Supplementary material for: First-Principles Assessment of ZnTe and CdSe as Prospective Tunnel Barriers at the InAs/Al Interface
Source: ACS Appl Mater Interfaces. 2025 Jan 13;17(3):5462–74. doi: 10.1021/acsami.4c17957 (PMC11758987; doi:10.1021/acsami.4c17957)
Supplement: Supplementary file 1 — am4c17957_si_001.pdf [file am4c17957_si_001.pdf]

Supporting Information
First Principles Assessment of ZnTe and CdSe as Prospective Tunnel Barriers at the InAs/Al Interface

Malcolm J. A. Jardine,[†] Derek Dardzinski,[†] Zefeng Cai,[†] Vladimir N. Strocov,[‡] Moïra Hocevar,[¶] Christopher J. Palmstrøm,[§,‖] Sergey M. Frolov,[⊥] and Noa Marom[*,†,#,@]

†*Department of Materials Science and Engineering, Carnegie Mellon University, Pittsburgh, PA 15213, USA*

‡*Paul Scherrer Institut, Swiss Light Source, CH-5232 Villigen PSI, Switzerland*

¶*Univ. Grenoble Alpes, CNRS, Grenoble INP, Institut Néel, 38000 Grenoble, France*

§*Materials Department, University of California-Santa Barbara, Santa Barbara, CA 93106, USA*

‖*Department of Electrical and Computer Engineering, University of California-Santa Barbara, Santa Barbara, CA 93106, USA*

⊥*Department of Physics and Astronomy, University of Pittsburgh, Pittsburgh, PA, 15260, USA*

#*Department of Physics, Carnegie Mellon University, Pittsburgh, PA 15213, USA*

@*Department of Chemistry, Carnegie Mellon University, Pittsburgh, PA 15213, USA*

E-mail: nmarom@andrew.cmu.edu

Bulk ZnTe and CdSe

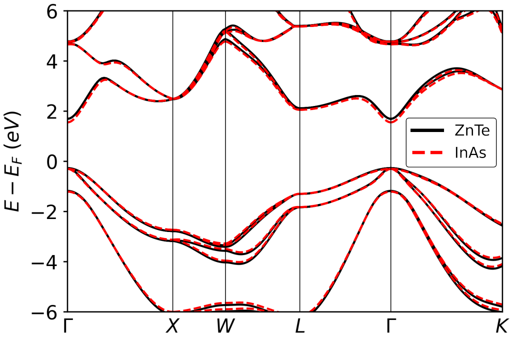

Figure S1: Comparison of the HSE band structures of ZnTe obtained with its lattice constant of (a) 6.101 Å of ZnTe and (b) 6.0584 Å of InAs. The difference in the lattice constant has no appreciable effect on the band structure other than a slight increase in band gap from 1.86 eV to 1.96 eV

To evaluate the sensitivity of the PBE+U(BO) results to the choice of U, we performed a series of PBE+U calculations for ZnTe and CdSe, varying $U_{\text{eff}}^{\text{Zn},3d}$ and $U_{\text{eff}}^{\text{Cd},4d}$ slightly around the optimal value (9.4 eV and 8.3 eV, respectively, which were determined using BO as shown in Figure S5.). We evaluated the change in the two terms of the BO objective function, the band gap change ΔGap, the band shape change $|\Delta\text{Band}|$, quantified via the RMSE of the first to fifth highest valence bands and the first to fifth lowest conduction bands, as well as the total change in the objective function $\Delta f(\vec{U})$ relative to the optimal U value. The results are presented in Figure S3 and Figure S4, as well as Table S1 and Table S2.

We find that for both materials a small change of up to about 0.5 eV in the U values does not lead to a significant change in the resulting band structure and in the BO objective function value. A change of about 1 eV leads to a noticeable difference. Our analysis reveals that while the band gap variation exhibits an approximately linear dependence on ΔU_{eff} within a small range, the energy shifts of individual states for both materials are often nonlinear or even non-monotonic with respect to changes in the Hubbard U. This complexity underscores the necessity of using an objective function and Bayesian optimization to quantify such intricate behavior and identify the optimal U value of interest.

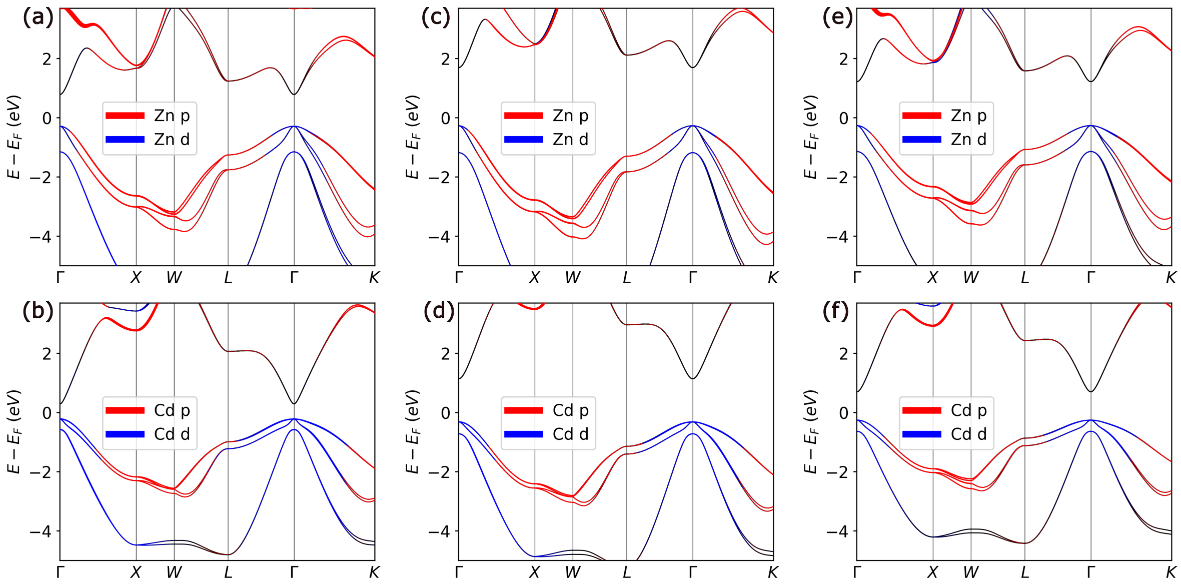

Figure S2: Orbital decomposition of the band structure obtained with different DFT functionals for ZnTe and CdSe: PBE band structures of (a) ZnTe and (b) CdSe; HSE band structures of (c) ZnTe and (d) CdSe; PBE + U(BO) band structures of (e) ZnTe and (f) CdSe. The contributions of the Zn/Cd p and Zn/Cd d states are indicated by the red and blue lines, respectively. The Hubbard U correction in PBE+U(BO) was applied to the Zn and Cd d states because they dominate the top of the valence band and the bottom of the conduction band for both materials.

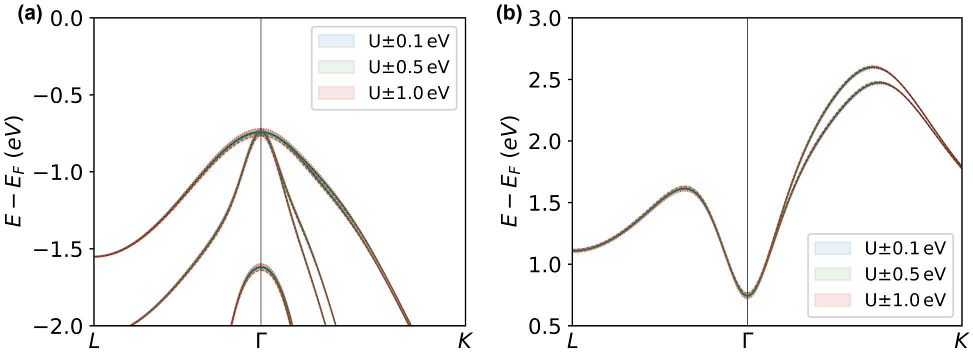

Figure S3: Sensitivity analysis of the PBE+U(BO) results for ZnTe: Band structures (a) around the VBM and (b) around the CBM, computed using $U_{\mathrm{eff}}^{\mathrm{Zn},3d}$ values varied around the optimal choice of 9.4 eV. Results with increased U_{eff} values (e.g., 9.4 eV + 0.1 eV) are plotted as dashed lines, while those with decreased U_{eff} values (e.g., 9.4 eV − 0.1 eV) are plotted as dash-dotted lines. The shaded areas represent the variation between the corresponding upper and lower bounds.

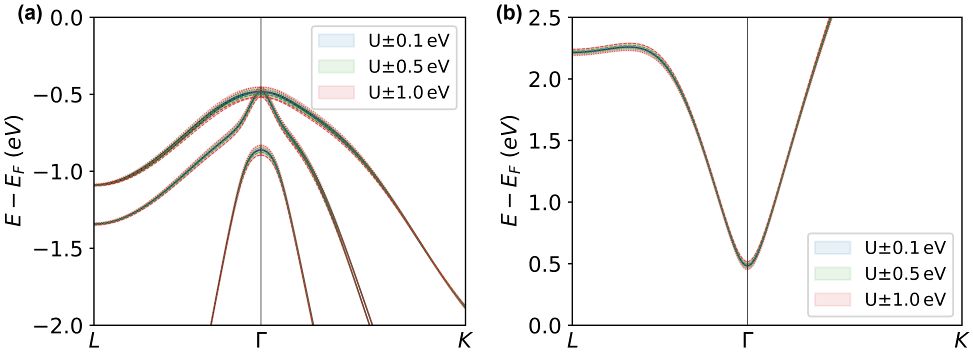

Figure S4: Sensitivity analysis of the PBE+U(BO) results for CdSe: Band structures (a) around the VBM and (b) around the CBM, computed using $U_{\mathrm{eff}}^{\mathrm{Cd},4d}$ values varied around the optimal choice of 8.3 eV. Results with increased U_{eff} values (e.g., 8.3 eV + 0.1 eV) are plotted as dashed lines, while those with decreased U_{eff} values (e.g., 8.3 eV − 0.1 eV) are plotted as dash-dotted lines. The shaded areas represent the variation between the corresponding upper and lower bounds.

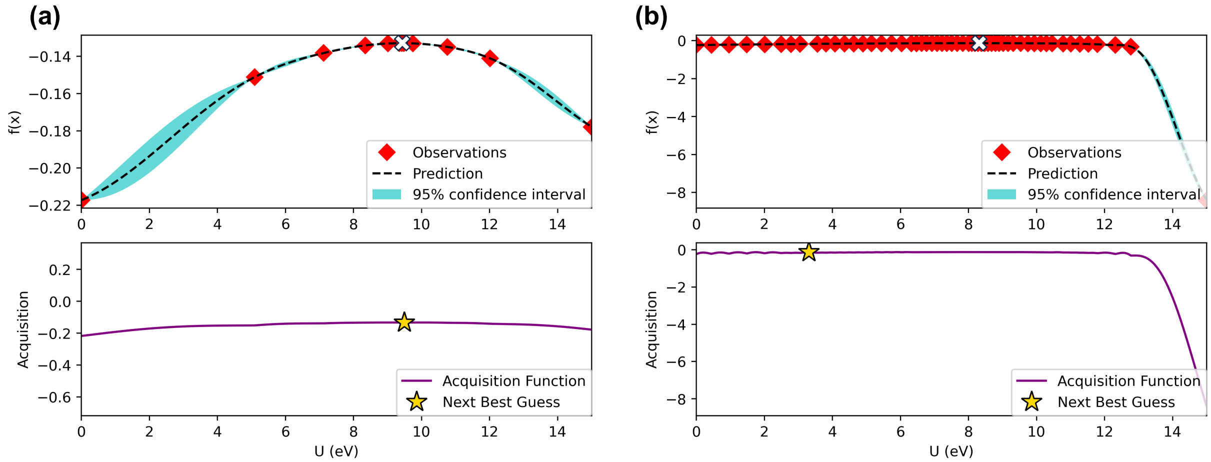

Figure S5: Gaussian process-predicted mean (top) and acquisition function (bottom) for the Bayesian optimization (BO) of the Hubbard U parameter for (a) ZnTe and (b) CdSe. White crosses denote the optimal U values, corresponding to the highest objective function values. For ZnTe the BO converged to the value of $U_{\mathrm{Zn}}^{3d,\mathrm{opt}} = 9.4$ eV after 12 steps. For CdSe the BO converged to the value of $U_{\mathrm{Cd}}^{4d,\mathrm{opt}} = 8.3$ eV after 56 steps. In this case, the predicted mean and acquisition function are quite flat over a wide range of U values.

Table S1: Hubbard U sensitivity analysis for ZnTe $3d$-orbital.

$\Delta U_{\text{eff}}^{\text{Zn},3d}$(eV)	$U_{\text{eff}}^{\text{Zn},3d}$(eV)	ΔGap (eV)	|ΔBand| (eV)	$\Delta f(\vec{U})$
−3.0	6.4	-0.1317	0.0571	0.0068
−1.0	8.4	-0.0447	0.0198	8.0e-4
−0.5	8.9	-0.0225	0.0100	2.0e-4
−0.3	9.1	-0.0135	0.0061	7.3e-5
−0.2	9.2	-0.0090	0.0041	3.3e-5
−0.1	9.3	-0.0045	0.0020	8.2e-6
Baseline	9.4	–	–	–
+0.1	9.5	0.0045	0.0020	8.3e-6
+0.2	9.6	0.0091	0.0041	3.3e-5
+0.3	9.7	0.0136	0.0061	7.5e-5
+0.5	9.9	0.0228	0.0103	2.1e-4
+1.0	10.4	0.0459	0.0207	8.5e-4
+3.0	12.4	0.1432	0.0665	0.0084

Table S2: Hubbard U sensitivity analysis for CdSe $4d$-orbital.

$\Delta U_{\text{eff}}^{\text{Cd},4d}$(eV)	$U_{\text{eff}}^{\text{Cd},4d}$(eV)	ΔGap (eV)	|ΔBand| (eV)	$\Delta f(\vec{U})$
−3.0	5.3	-0.1730	0.0735	0.0115
−1.0	7.3	-0.0612	0.0272	0.0014
−0.5	7.8	-0.0312	0.0140	3.9e-4
−0.3	8.0	-0.0189	0.0085	1.4e-4
−0.2	8.1	-0.0127	0.0057	6.5e-5
−0.1	8.2	-0.0064	0.0029	1.6e-5
Baseline	8.3	–	–	–
+0.1	8.4	0.0064	0.0029	1.7e-5
+0.2	8.5	0.0129	0.0059	6.8e-5
+0.3	8.6	0.0195	0.0089	1.5e-4
+0.5	8.8	0.0329	0.0151	4.4e-4
+1.0	9.3	0.0678	0.0315	0.0019
+3.0	11.3	0.2474	0.1218	0.0264

Comparison to ARPES for ZnTe

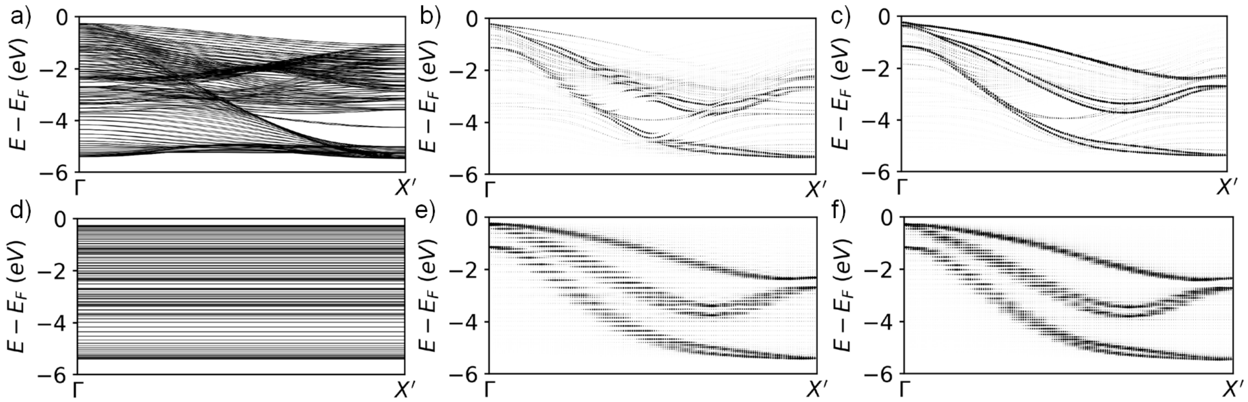

Figure S6: Band structure unfolding of a ZnTe slab: (a) Raw data along the surface-parallel $\Gamma - K|U' - X'$ path, showing a spaghetti-like band structure. Bulk-unfolded band structures along the surface-parallel $\Gamma - K|U' - X'$ path for slab models with (b) 30 layers and (c) 50 layers. With increasing slab thickness the unfolded band structure becomes more well-defined and bulk-like, and can be sampled infinitely. (d) Raw data along the surface-perpendicular $\Gamma - K|U' - X'$ path, showing the effect of quantum confinement in the perpendicular direction. Bulk-unfolded band structures along the surface-perpendicular $\Gamma - K|U' - X'$ path for slab models with (e) 30 layers and (f) 50 layers. Increasing the slab thickness reduces the quantum confinement and the unfolded band structure becomes more well-defined and bulk-like in this case, too. For a 3D periodic bulk crystal the two paths are symmetry-equivalent, but this is not the case for a slab model.

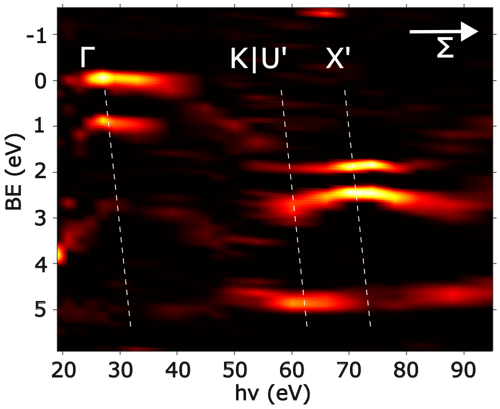

Figure S7: ARPES data adapted with permission from "Angle-resolved photoelectron spectroscopy study of the surface electronic structure of ZnTe(110)" by H. Qu *et al.*, Phys. Rev. B, 43, 9843 (1991); Copyright (1991) by the American Physical Society.[1]

Surface matching for bilayer interfaces

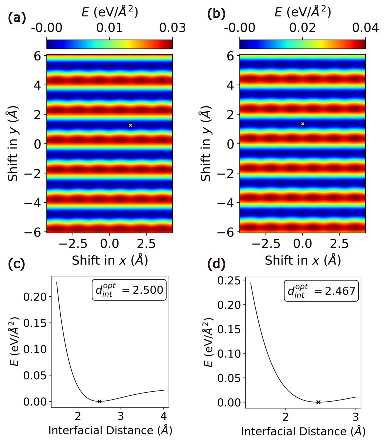

Figure S8: Interface construction for semiconductor/Al interfaces. Potential energy change as a function of the registry between the film and substrate with the minimum set to zero for (a) CdSe/Al and (b) ZnTe/Al. The minimum is indicated by a cross. Binding energy curve as function of interfacial distance for (c) CdSe/Al and (d) ZnTe/Al.

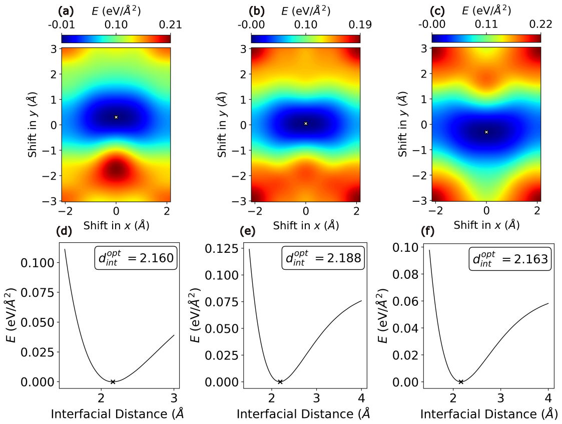

Figure S9: Interface construction for semiconductor/semiconductor interfaces. Potential energy change as a function of the registry between the film and substrate with the minimum set to zero for (a) InAs/ZnTe, (b) InAs/CdSe, and (c) ZnTe/CdSe. The minimum is indicated by a cross. Binding energy curve as function of interfacial distance for (d) InAs/ZnTe, (e) InAs/CdSe, and (f) ZnTe/CdSe.

Semiconductor interfaces

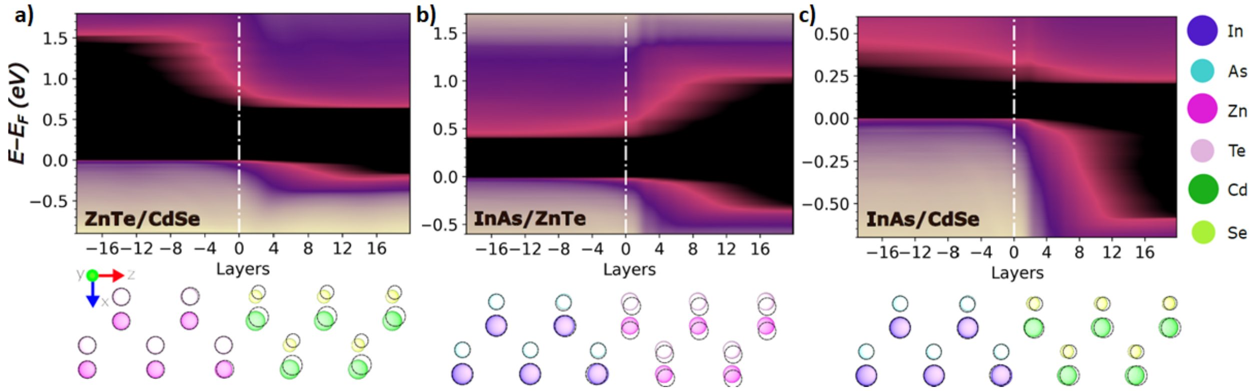

Figure S10: Electronic structure of unrelaxed semiconductor interfaces: Density of states as a function of position in the (a) ZnTe/CdSe, (b) InAs/ZnTe and (c) InAs/CdSe interfaces before relaxation. The atomic layers are numbered based on distance from the interface, which is located at zero. Illustrations of the structures are also shown with the relaxed atom positions shown as dashed circles on top of the unrelaxed structures. We find that small changes in atomic positions have a dramatic effect on the band alignment.

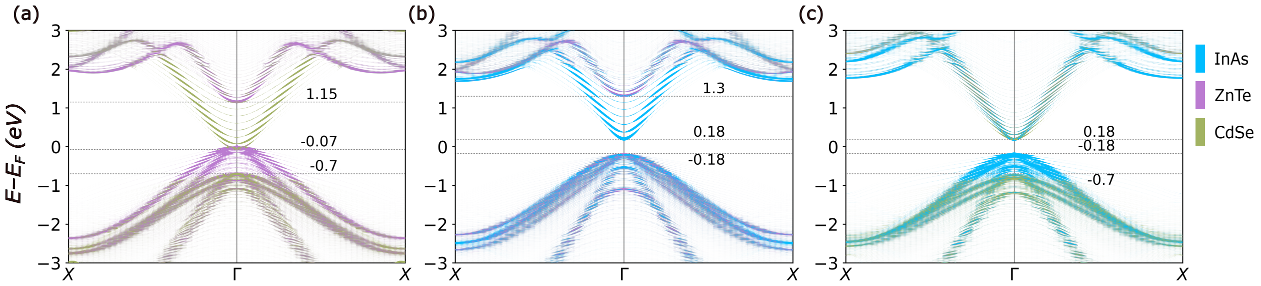

Figure S11: Electronic band structure of semiconductor-semiconductor bilayer interfaces: Element projected band structures of the (a) ZnTe/CdSe, (b) InAs/ZnTe and (c) InAs/CdSe interfaces, with bands originating from CdSe colored in green, bands originating from ZnTe colored in purple, and bands originating from InAs colored in light blue.

Semiconductor/Al interfaces

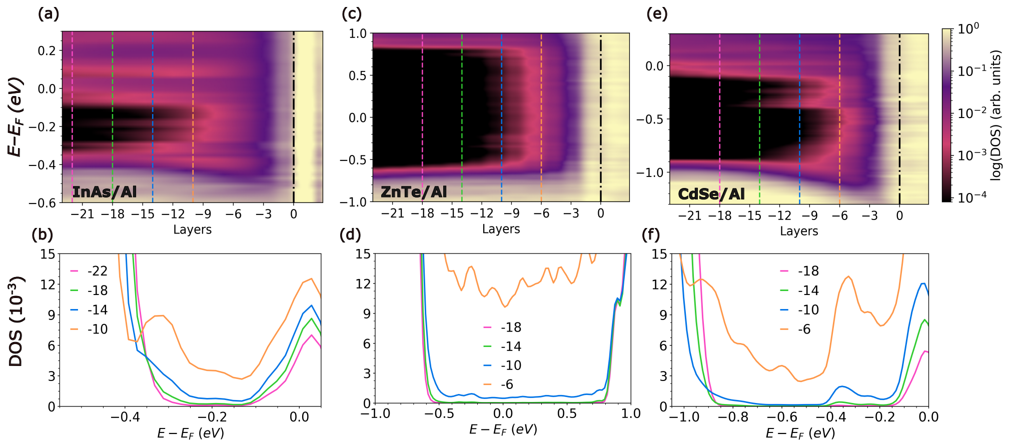

Figure S12: Electronic structure of semiconductor-Al bilayer interfaces: Density of states in the (a) InAs/Al, (c) ZnTe/Al and (e) CdSe/Al interfaces as a function of position. The atomic layers are numbered based on distance from the interface, which is located at zero. Local density of states for selected layers in the (b) InAs/Al, (d) ZnTe/Al and (f) CdSe/Al interfaces, indicated by dashed lines in the same colors in panels (a), (c), and (e), respectively. In InAs MIGS persist up to 18 layers from the interface. In ZnTe the MIGS decay more rapidly, within about 10 layers. In CdSe the MIGS decay within about 14 layers.

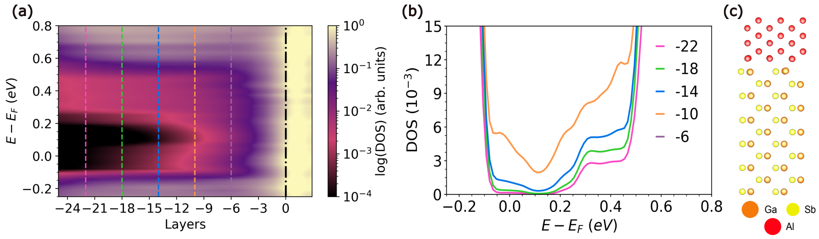

Figure S13: Electronic structure of the GaSb/Al interface: (a) Density of states as a function of position across the interface. The atomic layers are numbered based on distance from the interface, which is located at zero. The Fermi energy is in the GaSb gap with a Schottky barrier of ∼0.25 eV. (b) Local density of states for selected layers, indicated by dashed lines in the same colors in panel (a). Similar to the InAs/Al interface some MIGS persist up to 18 layers from the Al. (c) Illustration of the interface structure, comprising 30 layers of GaSb and 8 layers of Al with an interfacial distance of 2.362 Å.

Schottky Barrier Height

The Schottky barrier height (SBH) is calculated using the potential alignment method.[2–4] First, the electrostatic potential, $V_{\text{electrostatic}}$, and its macroscopic average, $\bar{V}_{\text{electrostatic}}$, are calculated for the three interfaces models, CdSe/Al, InAs/Al, and ZnTe/Al, as well as for the bulk systems Al, CdSe, InAs, and ZnTe, all subjected to the same strain conditions as the corresponding interfaces. The electrostatic potential is determined within DFT, defined as:

$$V_{\text{electrostatic}}(\mathbf{r}) = V_{\text{ionic}}(\mathbf{r}) + V_{\text{hartree}}(\mathbf{r}) = V_{\text{ionic}}(\mathbf{r}) + \int \frac{n(\mathbf{r}')}{|\mathbf{r}-\mathbf{r}'|} \mathrm{d}\mathbf{r}', \tag{1}$$

where $V_{\text{ionic}}(\mathbf{r})$ is the ionic potential as modeled by the pseudopotentials, and $V_{\text{hartree}}(\mathbf{r})$ is the Hartree potential.

The macroscopic average, $\bar{V}_{\text{electrostatic}}$, is obtained as the equally weighted moving average of $V_{\text{electrostatic}}$ over the z-axis as:

$$\bar{V}_{\text{electrostatic}}(z) = \frac{1}{L}\int_{z-L/2}^{z+L/2} \langle V_{\text{electrostatic}}\rangle_{xy}(z')\,\mathrm{d}z', \tag{2}$$

where $\langle V_{\text{electrostatic}}\rangle_{xy}$ is the planar average of $V_{\text{electrostatic}}$ over the xy-plane:

$$\langle V_{\text{electrostatic}}\rangle_{xy}(z) = \iint V_{\text{electrostatic}}(x,y,z)\,\mathrm{d}x\mathrm{d}y \Big/ \iint \mathrm{d}x\mathrm{d}y, \tag{3}$$

and L corresponds to the oscillation period along the z-direction at the center of the metal or semiconductor slab, typically a multiple of the interlayer distance.

The p-type and n-type SBH, ϕ_{p} and ϕ_{n}, are then calculated as follows:

$$\phi_{\text{p}} = E_{\text{Fermi}} - (E_{\text{VBM}} + \Delta\bar{V}), \tag{4}$$

$$\phi_{\text{n}} = E_{\text{g}} - \phi_{\text{p}}, \tag{5}$$

where $\Delta\bar{V}$ is the average electrostatic potential difference across the interface, defined as:

$$\Delta\bar{V} = \bar{V}^{\text{semiconductor}}_{\text{electrostatic}} - \bar{V}^{\text{metal}}_{\text{electrostatic}}\,, \tag{6}$$

which is always positive in our three interface models (the semiconductors exhibit a higher average potential). Here, the Fermi energy and band edges are calibrated using the bulk systems, such that E_{Fermi} is the metal Fermi level referenced to the average electrostatic potential of the bulk metal, E_{VBM} is the valence band maximum (VBM) referenced to the averaged electrostatic potential of the bulk semiconductor. E_{g} is the band gap of the bulk systems.

The optimized semiconductor/Al interfaces are used for SBH calculation, enabling atomic positions and interlayer spacing to adjust, rebalancing the charge and ensuring the convergence of the electrostatic potential distribution. In all of our models, we achieve convergence of $\Delta\bar{V}$ within 0.01 eV, as shown in Figure S14.

For all three interface systems, on both sides of the slab, the planar-averaged electrostatic potential converges to a well-defined, constant plateau, which corresponds to the vacuum electrostatic level, within approximately 7 Å. We note that the small fluctuation on the top right of is induced by the dipole correction.[5] It arises from the asymmetric nature of the slab and cannot be eliminated regardless of the vacuum layer thickness. Because the electrostatic potential converges much faster than the electronic structure with respect to the vacuum thickness, we do not recommend using the electrostatic potential as a convergence metric.

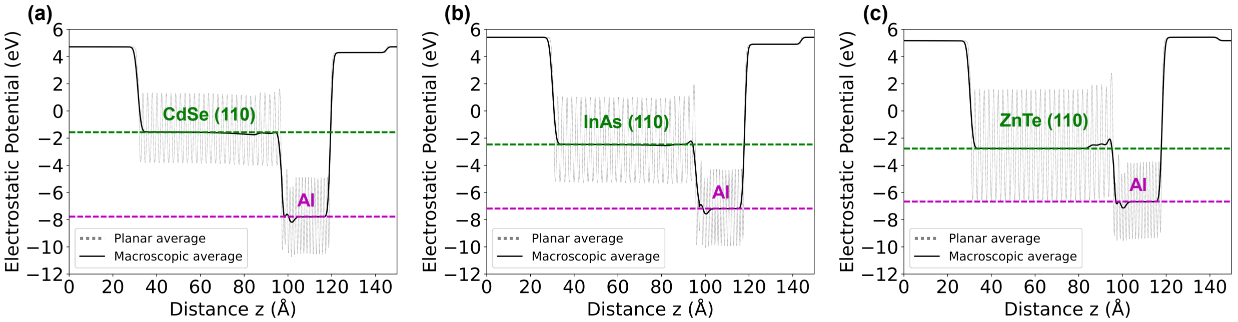

Figure S14: Electrostatic potential and corresponding macroscopic average for the three interface models of (a) CdSe/Al, (b) InAs/Al, and (c) ZnTe/Al. Grey dashed lines are the xy-planar average of the electrostatic potential along the z-axis. The solid black lines are the macroscopic (moving) average of the planar average. Green (purple) dashed line represent the utilized average potential value for the SM (Al) in the interface model which is used to calculate the average potential difference, $\Delta\bar{V}$, across the interface to determine the Schottky barrier height via the potential alignment method.

Table S3: Calibration of the Fermi level and valence band maximum (VBM) using the macroscopic average electrostatic potential for bulk Al, CdSe, InAs, and ZnTe under the same strain conditions as in the interface models.

	Al	CdSe	InAs	ZnTe
E_{Fermi}	7.9328	-	-	-
E_{VBM}	-	0.8035	2.8899	3.3974
$\bar{V}_{\text{electrostatic}}$	-0.0035	0.0010	0.0005	0.0005
Adj. E_{Fermi}	7.9363	-	-	-
Adj. E_{VBM}	-	0.8025	2.8894	3.3969

Table S4: Calculation of p-type and n-type Schottky barrier heights, ϕ_{p} and ϕ_{n}, for CdSe/Al, InAs/Al, and ZnTe/Al, using the potential alignment method. A negative value for the n-type Schottky barrier height indicates that the metal's Fermi level is located within the semiconductor's conduction band, signifying the absence of a Schottky barrier.

	CdSe/Al	InAs/Al	ZnTe/Al
Metal E_{Fermi}	7.9363	7.9363	7.9363
Semiconductor E_{VBM}	0.8025	2.8894	3.3969
Semiconductor E_{g}	0.9575	0.3154	1.4847
Average potential difference $\Delta\bar{V}$	6.19	4.68	3.91
$\phi_{\text{p}} = E_{\text{Fermi}} - (E_{\text{VBM}} + \Delta\bar{V})$	0.9438	0.3669	0.6294
$\phi_{\text{n}} = E_{\text{g}} - \phi_{\text{p}}$	0.0137	-0.0515	0.8553

Tri-layer interfaces

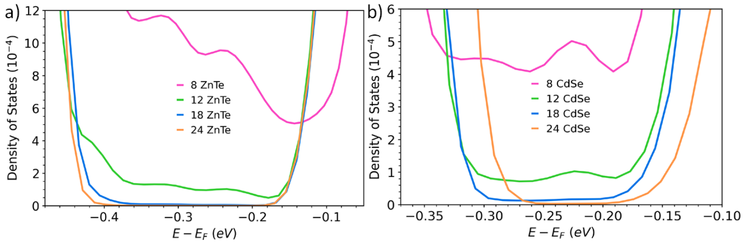

Figure S15: Density of states in the second InAs layer from the interface (layer -2) as a function of the number of (a) ZnTe and (b) CdSe barrier layers. With more than 16 atomic layers of either ZnTe or CdSe no MIGS are seen in the InAs.

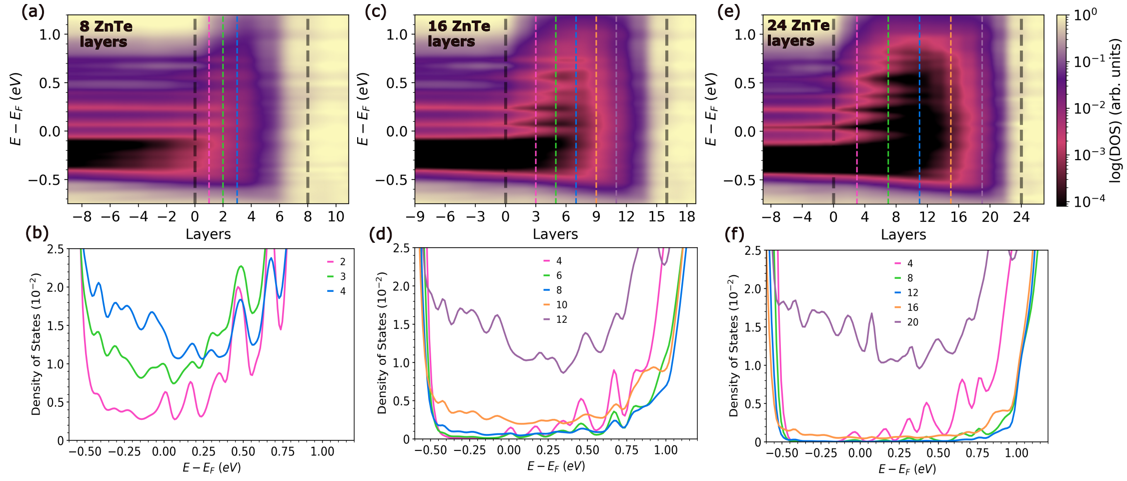

Figure S16: Evolution of the ZnTe band gap with the barrier thickness in InAs/ZnTe/Al interfaces: Density of states as a function of distance from the interface for (a) 8, (c) 16 and (e) 24 ZnTe barrier layers. The atomic layers are numbered based on distance from the interface, which is located at zero. (b), (d), (f) Local density of states for selected layers, indicated by dashed lines in the same colors in panels (a), (c), and (e), respectively. Only with 24 layers the ZnTe band gap approaches the expected value in the middle of the film.

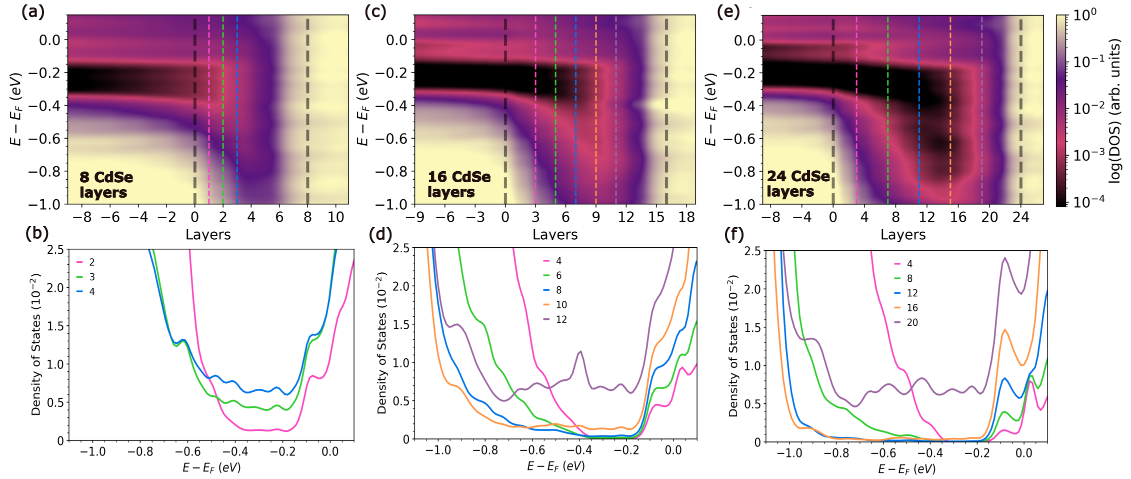

Figure S17: Evolution of the CdSe band gap with the barrier thickness in InAs/CdSe/Al interfaces: Density of states as a function of distance from the interface for (a) 8, (c) 16 and (e) 24 CdSe barrier layers. The atomic layers are numbered based on distance from the interface, which is located at zero. (b), (d), (f) Local density of states for selected layers, indicated by dashed lines in the same colors in panels (a), (c), and (e), respectively. Only with 24 layers the ZnTe band gap approaches the expected value in the middle of the film.

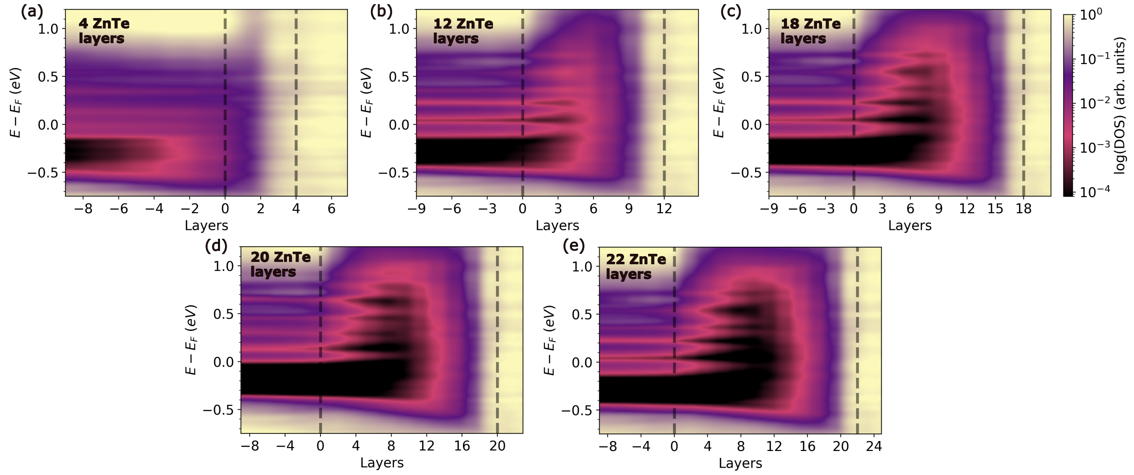

Figure S18: Electronic structure of InAs/ZnTe/Al tri-layer interfaces: Density of states as a function of distance from the interface for (a) 4, (b) 12, (c) 18, (d) 20, and (e) 22 ZnTe barrier layers. The atomic layers are numbered based on distance from the interface, which is located at zero. Interfaces are indicated by dashed lines.

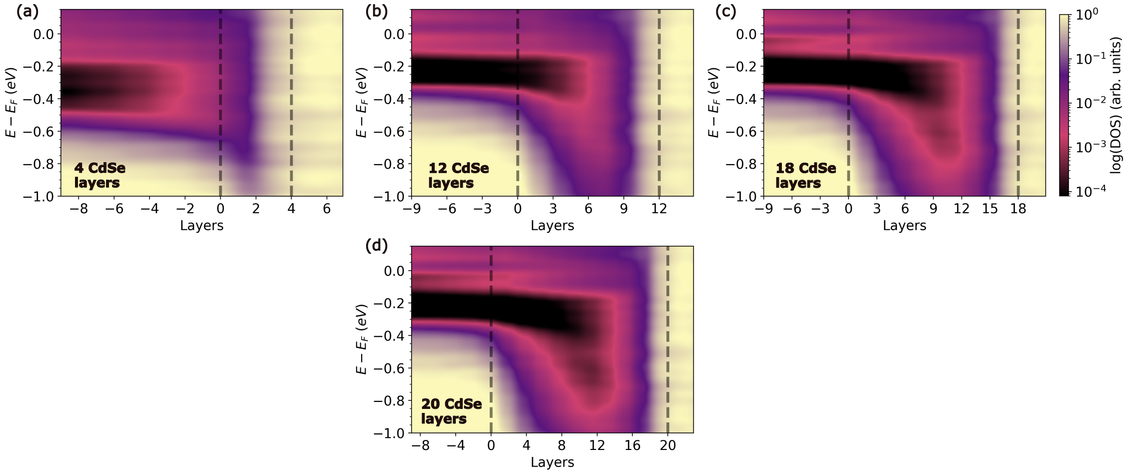

Figure S19: Electronic structure of InAs/CdSe/Al tri-layer interfaces: Density of states as a function of distance from the interface for (a) 4, (b) 12, (c) 18, and (d) 20 CdSe barrier layers. The atomic layers are numbered based on distance from the interface, which is located at zero. Interfaces are indicated by dashed lines.

References

(1) Qu, H.; Kanski, J.; Nilsson, P. O.; Karlsson, U. O. Angle-resolved photoelectron spectroscopy study of the surface electronic structure of ZnTe(110). *Phys. Rev. B* **1991**, *43*, 9843–9850.

(2) Delaney, K. T.; Spaldin, N. A.; Van de Walle, C. G. Theoretical study of Schottky-barrier formation at epitaxial rare-earth-metal/semiconductor interfaces. *Phys. Rev. B* **2010**, *81*, 165312.

(3) Weston, L.; Tailor, H.; Krishnaswamy, K.; Bjaalie, L.; Van de Walle, C. G. Accurate and efficient band-offset calculations from density functional theory. *Computational Materials Science* **2018**, *151*, 174–180.

(4) Nangoi, J. K.; Palmstrøm, C. J.; Van de Walle, C. G. First-principles studies of Schottky barriers and tunneling properties at Al(111)/Si(111) and $CoSi_2$(111)/Si(111) interfaces. *Phys. Rev. B* **2024**, *110*, 035302.

(5) Neugebauer, J.; Scheffler, M. Adsorbate-substrate and adsorbate-adsorbate interactions of Na and K adlayers on Al (111). *Physical Review B* **1992**, *46*, 16067.
